# Cross‐Sectional Associations Between Exposure to Commercial Milk Formula Marketing, Beliefs About Its Use, and Socioeconomic Position Among Pregnant Women and Mothers in the UK

**DOI:** 10.1111/mcn.70022

**Published:** 2025-03-24

**Authors:** Martha Athanasiadou, Florence Sheen, Andrea D. Smith, Clare Llewellyn, Rana Conway

**Affiliations:** ^1^ Research, Department of Behavioural Science and Health University College London London UK; ^2^ School of Sport, Exercise and Health Sciences, National Centre for Sport and Exercise Medicine Loughborough University Loughborough UK; ^3^ MRC Epidemiology Unit University of Cambridge Cambridge UK

**Keywords:** breastfeeding, breastmilk substitute, commercial milk formula, infant feeding, infant formula, marketing, socioeconomic position

## Abstract

International provisions are in place to restrict marketing practices that idealise the use of commercial milk formula (CMF) and discourage breastfeeding. In high‐income countries, women of lower socioeconomic position (SEP) are less likely to breastfeed. This study aimed to characterise the nature of exposure to CMF marketing in the UK, the extent to which women hold positive beliefs about CMF and examine the relationship between exposure to CMF marketing, holding positive beliefs about CMF and SEP. Data on these topics were collected for 1052 pregnant women and mothers of children under 18 months of age between February 2020 and February 2021. Participants were assigned a ‘CMF marketing score’ according to the number of locations where they recalled seeing advertisements, engaging with companies or receiving promotional activity. The extent to which women held positive beliefs about CMF (‘CMF positivity score’) was determined by level of agreement with 17 statements. Principal component analysis, analyses of covariance and regression analyses were applied. Every woman reported exposure to CMF marketing from multiple channels. CMF marketing score did not vary across SEP groups (*p* = 0.342). Women of lower and middle SEP held stronger CMF positive beliefs than women of higher SEP, including ‘Breastfeeding and formula feeding provide a baby with the same health benefits’ (*p* < 0.005). CMF marketing score was not associated with CMF positivity score. Marketing suggesting CMF provides benefits similar to, or greater than, breastfeeding should be restricted to help mitigate current inequalities in infant feeding practices.

## Summary

- Every woman in this UK sample reported exposure to commercial milk formula (CMF) marketing, including frequently seeing advertisements on TV and receiving pop‐up advertisements on social media.
- Women of lower and middle socioeconomic position held stronger beliefs that CMF provides the same or greater benefits than breastfeeding than women of higher SEP.
- No association was found between the number of forms of CMF marketing women were exposed to and holding more positive beliefs about CMF.
- To support a reduction in infant feeding inequalities, detailed legislation is needed to restrict marketing that suggests CMF provides equal or superior benefits to breastfeeding.

## Graphical abstract

All women in this UK study reported exposure to formula milk marketing. Women of lower and middle socioeconomic position (SEP) were more likely than those of higher SEP to believe formula provides equal or greater benefits than breastfeeding. Tighter legislation is needed to restrict marketing suggesting formula is breastmilk equivalent/superior.

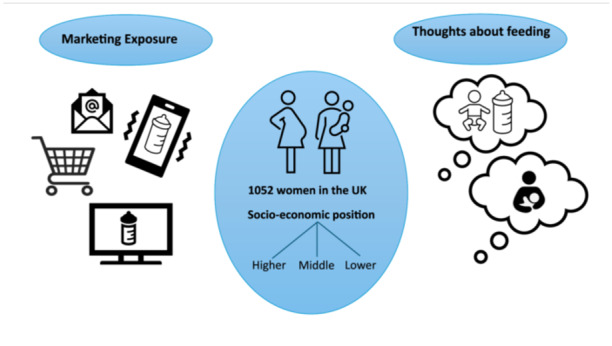

## 1 Introduction

In high‐income counties, women of lower socioeconomic position (SEP) are less likely to breastfeed than women of higher SEP (Victora et al. 2016). The World Health Organization (WHO) recommends exclusive breastfeeding for the first 6 months of an infant's life and continuation of breastfeeding until at least 2 years of age once complementary foods are introduced (World Health Organization 2003). However, in England half of infants (52.2%) receive no breastmilk beyond the first 6–8 weeks of life (Office for Health Improvement and Disparities 2023).

Inequalities in infant feeding practices have been shown across a range of SEP indicators. For example, in England approximately 78% of women living in the poorest neighbourhoods ever breastfeed, compared with 92% in the richest neighbourhoods (Quigley et al. 2023). Research from high‐income countries found that breastfeeding is more common among women who have higher educational attainment and belong to more wealthy households than those with fewer years of formal education and less household wealth (Victora et al. 2016). Similarly, mothers in higher managerial or professional occupations (e.g., human resource director, surgeon) are four times more likely to initiate breastfeeding compared to mothers in routine occupations (e.g., catering assistant, farm worker) and those in higher managerial or professional occupations are also more likely to exclusively breastfeed their baby at 4 months of age (Kelly and Watt 2005). Studies using a combination of SEP indicators have found that mothers of middle‐to‐higher SEP are 1.5–2 times more likely to meet recommendations to maintain exclusive breastfeeding for 6 months compared to those of lower SEP (Moran‐Lev et al. 2021; Ramiro González et al. 2018).

Reasons for socioeconomic differences in breastfeeding rates are complex. Infant feeding practices are determined by individual, socioeconomic and cultural factors, such as employment conditions and healthcare services, as well as marketing of breastmilk substitutes (Hernández‐Cordero et al. 2022; Rollins et al. 2016). Lower breastfeeding rates amongst mothers of lower SEP are in part due to situational barriers, such as a greater need to return to work, less flexible working arrangements (Heinig et al. 2006) lower self‐efficacy (Mitra et al. 2004) and limited access to help for breastfeeding problems (McIntyre et al. 2001). Beliefs about commercial milk formula (CMF) and breastfeeding may also play a role. Research with lower‐income mothers in high‐income countries shows that negative beliefs about breastfeeding are common, such as believing that breastfeeding is difficult (Guttman and Zimmerman 2000), that infants' hunger will not be satisfied from breastmilk (Heinig et al. 2006) or feeling that breastfeeding is embarrassing (McCann et al. 2007). However, variation in such beliefs across the socioeconomic spectrum is unclear.

There is concern that the widespread marketing of CMF shapes mothers' beliefs and infant feeding decisions and thereby contributes to low breastfeeding rates globally (McFadden et al. 2016; Rosenberg et al. 2008; Zhang et al. 2013). New parents are particularly vulnerable because of uncertainty over their new roles and their wish to do the ‘best’ for their child (Hastings et al. 2020; Henshaw et al. 2018). Parents who believe the marketing claims promoting the benefits of CMF are more likely to serve it to their child (Romo‐Palafox et al. 2020). Therefore, understanding parents' beliefs about CMF, including segmenting parents according to their parenting styles, anxieties and aspirations is a key part of CMF marketers' activity (Hastings et al. 2020; Rollins et al. 2023). Longitudinal analysis of magazine advertisements for CMF in the United States (US) has shown that in years when advertising frequency increased, breastfeeding rates over the following year tended to decrease (Foss and Southwell 2006). Also, mothers in the US who recall exposure to CMF marketing from print media or websites have been found more likely to intend to use CMF or use it earlier and less likely to initiate breastfeeding than mothers who do not recall seeing such marketing (Zhang et al. 2013). Therefore, a better understanding of the impact of CMF marketing is key to allowing parents to make informed choices about the way they feed their children free from commercial influence.

This study aimed to characterise the nature of exposure to CMF marketing, the extent to which women hold positive beliefs about CMF and associations with SEP in the UK. Secondly, the study aimed to examine the association between exposure to CMF marketing and holding positive beliefs about CMF. We hypothesised that individuals who reported higher exposure to CMF marketing would hold more positive beliefs, and that individuals of lower SEP would hold more positive beliefs than individuals of medium or higher SEP.

## 2 Methods

### 2.1 Design

This study employed a survey design using researcher‐led interviews (in‐person or online) to collect data from women living in the UK. A communication and behaviour change company (M&C Saatchi World Services) was commissioned by WHO to collect these data as part of a multi‐country study. Questions used to assess exposure to CMF marketing and beliefs about CMF were developed by M&C Saatchi World Services. These had been informed by their marketing analysis (World Health Organization, United Nations Children's Fund ‎UNICEF‎ & M&C Saatchi World Services 2022). Analysis and interpretation of the data were conducted independently by the authors.

### 2.2 Participants

To be eligible for inclusion, participants had to be 18 years or older, fluent in English, at least 3 months pregnant or the mother of a child aged 0–18 months of age. Women were excluded from participating if either they, a member of their family or a close friend worked in a market research, advertising, or CMF company, or if they had attended a group discussion or interview related to ‘mother‐and‐baby’ issues in the past 12 months, or if they had a medical condition that would prevent, or had prevented, breastfeeding. The terms women, mothers and breastfeeding are used for brevity throughout the manuscript, but it is acknowledged that not all people who breastfeed/chestfeed identify as women.

Participants were recruited between February 2020 and February 2021 from London and the South of England, Midlands, North of England and Scotland using online convenience methods, including research panels and targeted advertising (street‐based, door‐to‐door, online and through healthcare facilities). Quota sampling was applied to recruit approximately equal numbers of participants according to three SEP groups (lower, middle, higher occupational status of main income earner). Quotas were also set on feeding behaviours, for those with children, to ensure inclusion of women who exclusively breastfeed, exclusively use CMF or use mixed feeding. Approximately equal number of participants were recruited from seven parenting groups (pregnant first‐time mother, pregnant multiparous mother, mother exclusively breastfeeding an infant 0–12 months of age, or mother feeding CMF to an infant/child aged 0–3, 4–6, 7–12 or 13–18 months of age).

### 2.3 Procedure

The survey was conducted via a questionnaire administered by an interviewer (Supporting Information S1). Initially, surveys were administered face‐to‐face in the respondent's home or in another convenient location. When COVID‐19 restrictions applied, the survey was conducted via Zoom or phone. Eligibility to participate was assessed using a 17‐item screener questionnaire, to confirm criteria for inclusion and quota sampling, before the full survey was administered. Each session lasted about 30 minutes. Computer‐Assisted Personal Interviewing software was used by the interviewer to record survey responses online. Written and verbal informed consent was collected by the interviewer.

### 2.4 Measures

#### 2.4.1 SEP

Participants were asked to identify which, out of seven predefined occupational groups, the main income earner in their household belonged to. The seven occupational groups were then collapsed into three SEP groups: lower (manual, casual worker, unemployed), middle (supervisory, skilled manual) and higher (professional, middle management) (Supporting Information S1). This was considered the most appropriate indicator of SEP for this analysis, rather than education or household income, as this was used for quota sampling and participant numbers were approximately equal in the three occupation‐based SEP groups.

#### 2.4.2 CMF Marketing Score

Participants were presented with a list of 33 different methods of CMF marketing (see results section). From the list, they were asked to indicate any of 16 locations, where they had frequently seen CMF marketing or advertising. They also indicated how they had engaged with CMF companies from 7 possible ways (e.g., participating in a competition run by a CMF company) and any of 10 CMF promotional activities they had received (e.g., free CMF samples in hospital). The sum of participants' scores could range from 0 to 33, with higher scores indicating exposure to a greater range of methods of CMF marketing. For example, if a participant had seen formula advertisements on TV and YouTube, received one free sample and followed a formula company on social media, they would score 4.

#### 2.4.3 CMF Positivity Score

Participants were presented with 17 belief statements about infant feeding (see results table) and asked to indicate ‘Disagree’, ‘Neither Agree nor Disagree’ or ‘Agree’ on a three‐point Likert scale. Answers were scored 0, 1 or 2 for statements expressing a positive belief about CMF, such as ‘formula is very like breastmilk’. Reverse scoring (2, 1 or 0) was applied to five statements showing positive beliefs about breastfeeding, such as ‘breastfed babies are healthier than formula fed babies’. Participants could also indicate ‘don't know/not applicable’ which was treated as missing data and was not coded. Participants with missing data were not included in analyses where summed scores were used. Scores for each answer were summed so that a higher CMF positivity score indicated more positive beliefs towards CMF (possible range 0–34).

### 2.5 Statistical Analyses

All analyses were conducted using IBM SPSS Statistics (version 27). Sociodemographic characteristics were analysed descriptively for participants within each SEP group. Mean CMF marketing scores and mean CMF positivity scores were examined for the total sample and for each SEP group.

Principal component analysis (PCA) was applied to identify underlying constructs (belief factors) within the 17 items (belief statements) making up the CMF positivity score. Varimax rotation was used with Kaiser normalisation. The number of components to be retained was determined based on the Kaiser's criterion (Kaiser 1960). For items loading to more than one factor, the factor with the higher absolute value of loading was chosen, unless factors with a lower loading were considered a better fit (Field 2017). Reliability of the belief factors identified in the PCA was evaluated using Cronbach's alpha coefficients of internal consistency. Differences between the three SEP groups' CMF marketing scores CMF and for each of the PCA‐derived belief factors were then examined using a one‐way analysis of covariance adjusted for respondent's age and parity. Both maternal age (Bjørset et al. 2018; Biro et al. 2014) and parity (Thulier and Mercer 2009) have been found to be strongly related to breastfeeding and thus were deemed appropriate as covariates. Sociodemographic factors such as ethnicity or income were not included as covariates to avoid overadjustment. For factors where Cronbach's alpha values indicated the scale was not acceptable to use (*α* < 0.70), the associated belief statements were included in a multivariate analysis of covariance (MANCOVA) to explore differences between the three SEP groups. Post hoc tests were conducted to explore differences between specific levels of SEP and a Bonferroni correction was applied. Linear regression was used to examine associations between CMF marketing score and each of the PCA‐derived belief factors. All regression models were adjusted for respondent's age and parity.

### 2.6 Ethical Statement

Ethical approval was granted by the Research Ethics Committee of Queen Mary University of London on 20 February 2020 (QMERC2019/61).

## 3 Results

In total, 1052 women aged 18–47 years participated, including 300 pregnant women and 752 mothers of children aged 0–18 months. Sample characteristics of the 1052 women are displayed in Table 1. Compared to women of higher SEP (based on occupational group), those of lower SEP tended to be younger, have completed fewer years of formal education, and have a lower household income. Participants of lower SEP were also more likely to be single, have at least one child in addition to the reference child and be full‐time mothers or unemployed.

**Table 1:** Sample characteristics for the overall sample and by socioeconomic position (SEP) group of pregnant women or women with a child < 18 months in the UK.

Characteristics	Overall sample (*n* = 1052)	Lower SEP (*n* = 330)	Middle SEP (*n* = 359)	Higher SEP (*n* = 363)
Income, *n* (%)^a^				
Less than £10,000	55 (5.2)	50 (15.2)	5 (1.4)	0 (0)
£10,000–£39,999	427 (40.6)	230 (69.7)	152 (42.3)	45 (12.4)
£40,000–£69,999	296 (28.1)	29 (8.8)	148 (41.2)	119 (32.8)
£70,000–£99,999	158 (15.0)	7 (2.1)	39 (10.9)	112 (30.9)
£100,000+	83 (7.9)	0 (0)	8 (2.2)	75 (20.7)
Prefer not to say	33 (3.1)	14 (4.2)	7 (1.9)	12 (3.3)
Child age^b^, *n* (%)				
Younger than 2 months	72 (9.6)	20 (8.5)	29 (11.3)	23 (8.8)
2–6 months	310 (41.2)	96 (40.9)	97 (37.7)	117 (45.0)
7–12 months	192 (25.5)	61 (26.0)	68 (26.5)	63 (24.2)
13+ months	178 (23.7)	58 (24.7)	63 (24.5)	57 (21.9)
Marital status, *n* (%)				
Single	129 (12.3)	81 (24.5)	31 (8.6)	17 (4.7)
Married/cohabitate	907 (86.2)	240 (72.7)	323 (90.0)	344 (94.8)
Divorced/separated	14 (1.3)	9 (2.7)	3 (0.8)	2 (0.6)
Prefer not to say	2 (0.2)	0 (0)	2 (0.6)	0 (0)
Maternity pay^c^, *n* (%)				
Statutory maternity pay	314 (33.8)	79 (34.6)	128 (37.0)	107 (30.1)
Maternity pay paid by employer	310 (33.3)	32 (14.0)	114 (32.9)	164 (46.1)
Not receiving	306 (32.9)	117 (51.3)	104 (30.1)	85 (23.9)
Work, *n* (%)				
Full‐time	231 (22.0)	26 (7.9)	94 (26.2)	111 (30.6)
Part‐time	179 (17.0)	53 (16.1)	79 (22.0)	47 (12.9)
Full‐time education	14 (1.3)	7 (2.1)	6 (1.7)	1 (0.3)
Full‐time mother/housewife or unemployed	251 (23.9)	172 (52.1)	51 (14.2)	28 (7.7)
Maternity leave	377 (35.8)	72 (21.8)	129 (35.9)	176 (48.5)
Parity^d^, *n* (%)				
None	542 (51.5)	151 (45.8)	187 (52.1)	204 (56.2)
One	337 (32.0)	93 (28.2)	121 (33.7)	123 (33.9)
Two	118 (11.2)	51 (15.5)	36 (10.0)	31 (8.5)
More than two	55 (5.2)	35 (10.6)	15 (4.2)	5 (1.4)
Age group, *n* (%)				
18–24	81 (7.7)	59 (17.9)	19 (5.3)	3 (0.8)
25–29	206 (19.6)	88 (26.7)	80 (22.3)	38 (10.5)
30–34	407 (38.7)	111 (33.6)	146 (40.7)	150 (41.3)
35–39	282 (26.8)	54 (16.4)	94 (26.2)	134 (36.9)
40+	76 (7.2)	18 (5.5)	20 (5.6)	38 (10.5)
Highest level of education, *n* (%)				
Primary or none	3 (0.3)	1 (0.3)	2 (0.6)	0 (0)
Secondary	277 (26.3)	180 (54.5)	77 (21.4)	20 (5.5)
Higher or post‐graduate	762 (72.4)	144 (43.6)	278 (77.4)	340 (93.7)
Other	10 (1.0)	5 (1.5)	2 (0.6)	3 (0.8)
Ethnicity, *n* (%)				
White	835 (79.4)	262 (79.4)	276 (76.9)	297 (81.8)
Mixed	63 (6.0)	25 (7.6)	25 (7.0)	13 (3.6)
Asian	73 (6.9)	21 (6.4)	31 (8.6)	21 (5.8)
Black	73 (6.9)	19 (5.8)	25 (7.0)	29 (8.0)
Other	8 (0.8)	3 (0.9)	2 (0.6)	3 (0.8)

^a^ Correlation between SEP and income is reported in Supporting Information S1.

^b^ Child age applies to mothers only (*n* = 752).

^c^ Maternity pay/benefits information missing for 122 women.

^d^ The number of children indicated refers to the number of children that a woman may have other than the one she is expecting or her 0–18‐month child.

Every participant in this study reported being exposed to CMF marketing (Table 2). Two thirds of women recalled frequently seeing CMF advertising on TV (67.6%) and almost half reported frequently seeing CMF marketing on social media (45.3%). One‐third had frequently seen in‐store marketing (33.5%) and one‐fifth reported frequently seeing advertising in magazines or newspapers (20.6%). When asked about engagement with CMF companies, nearly half of women reported some form of active engagement (45.9%), including one in five using information from a CMF company (22.7%) and one in five signing up for a baby club run by a CMF company (22.1%). With regard to promotional activities from CMF companies, nearly half of women reported receiving pop‐up advertisements on social media (47.1%), almost one‐third had been invited to join a baby club run by a company (31.1%) and one quarter had received a promotion for a discount (26.8%). Overall, women reported being exposed to a mean of six (6.43 ± 3.76) different forms of CMF marketing or advertising in the past year. No difference in CMF marketing scores was found across the three SEP groups, *p* = 0.342 [lower (*M* = 6.68, SD = 3.76), middle (*M* = 6.24, SD = 3.72), higher (*M* = 6.40, SD = 3.81)].

**Table 2:** Participants' self‐reported exposure to commercial milk formula (CMF) marketing over the past year, overall and by socioeconomic position (SEP) group.

Exposure to CMF marketing	Total sample (*n* = 1052)	Lower SEP (*n* = 330)	Middle SEP (*n* = 359)	Higher SEP (*n* = 363)
Location where marketing or advertising frequently seen^a^, %				
TV	67.6	67.0	65.2	70.5
Radio	1.8	2.7	1.4	1.4
YouTube	17.7	15.5	18.1	19.3
Company website e.g., a specific brand website	13.4	13.6	12.8	13.8
Social media e.g., Facebook, Instagram, celebrity or mummy blogger	45.3	44.8	45.1	46.0
Online discussion forum or chat room	12.9	13.6	11.7	13.5
Another website	6.4	7.0	5.8	6.3
Mother's club or group online	17.5	16.1	17.0	19.3
In an email	13.4	13.9	13.6	12.7
In the post	7.3	10.0	6.1	6.1
A hospital or clinic	12.8	17.0	10.9	11.0
Magazine or newspaper	20.6	18.5	17.8	25.3
Billboard e.g., on the highway or roadside	3.8	6.1	2.8	2.8
Supermarket/shop/market—in store	33.5	37.9	30.9	32.0
Supermarket/shop—online	14.9	18.8	11.4	14.9
Other	10.9	10.6	8.4	13.8
Engagement with companies^b^, %				
Follow a formula company on social media	11.4	16.1	11.4	7.2
Use an app from a formula company	3.4	6.1	1.7	2.8
Participate in any baby competitions run by formula companies	7.5	8.5	8.6	5.5
Use any information from formula companies	22.7	21.8	22.6	23.7
Registered for updates/newsletter from a formula company	15.9	13.0	18.9	15.4
Signed up/registered for a baby club run by a formula company	22.1	20.9	22.6	22.9
Followed a person on Instagram or Facebook for information on formula feeding	8.0	10.9	7.5	5.8
None of these	54.1	53.3	54.0	54.8
Promotional activity received^c^, %				
Information or contact from a formula company by email, post, phone, or text message that you have not requested	13.8	16.4	12.9	12.5
Promotion for formula discount	26.8	29.9	25.8	25.1
Free samples of formula milk in hospital	17.5	19.1	15.5	17.9
Free sample of formula milk outside hospital	3.7	4.9	2.8	3.6
Free bottles or teats	25.0	19.1	28.4	27.0
Any other gifts from a formula company e.g., a toy, bag, or clothing	16.6	16.1	19.5	14.1
An invite to join a ‘baby club’ run by a formula company	31.1	28.7	32.8	31.8
An invite to a competition from a formula company or from a shop	13.5	14.9	10.9	14.6
An invite from a formula company to participate in research/survey/interview	5.2	4.9	5.6	5.0
Received a pop‐up advert on Facebook or other social media	47.1	51.4	46.0	44.2

^a^ Derived from the question: ‘In which of these locations have you frequently seen any type of marketing or advertising for formula brands?’ Responses were provided as multiple selections from a list of predefined choices. There was no maximum number of allowed selections.

^b^ Derived from responses to the question: ‘Do/have you done any of the following?’ Responses were provided as multiple selections from a list of predefined choices. There was no maximum number of allowed selections.

^c^ Derived from responses to the question: ‘Have you ever received any of the following?’ Responses to each promotional activity listed were provided as ‘Yes’, ‘No’ or ‘Do not know/Not Applicable’.

Many women held positive beliefs about CMF (Table 3). One in four women (28.2%) agreed with the statement ‘breastfeeding and formula feeding provide a baby with the same health benefits’, including 35.7% of the lower SEP group and 20.8% of the higher SEP group. Following the PCA of belief statements, a six‐factor solution was considered most appropriate. Factor names were given to reflect the underlying construct within each factor. Factor names and factor loadings after rotation are displayed in Table 4. However, with the exception of ‘Formula keeps babies contented’ (*α* = 0.71; *n* = 2 items), no belief factors obtained an acceptable Cronbach *α* value ≥ 0.70 (*α*s < 0.55, see Table 4). Therefore, a MANCOVA was deemed more suitable and was subsequently applied for the remaining 15 belief statements with a Bonferroni correction applied.

**Table 3:** Women's beliefs about infant feeding, overall and by socioeconomic position (SEP) group.

	Total sample (*N* = 1052)		Lower SEP (*N* = 330)		Middle SEP (*N* = 359)		Higher SEP (*N* = 363)	
Belief statements	Women agreeing with statement, %	Women completing question, *n* (%)	Women agreeing with statement, %	Women completing question, *n* (%)	Women agreeing with statement, %	Women completing question, *n* (%)	Women agreeing with statement, %	Women completing question, *n* (%)
1 Formula feeding is the better choice if the mother plans to go back to work	30.6	1040 (98.9)	39.8	327 (99.1)	29.0	355 (98.9)	23.7	358 (98.6)
2 Breastfeeding is best for your baby^a^	75.0	1049 (99.7)	67.8	329 (99.7)	74.6	358 (99.7)	82.0	362 (99.7)
3 Formula fed babies grow better than breastfed babies	4.7	1015 (96.5)	5.0	323 (97.9)	4.9	344 (95.8)	4.3	348 (95.9)
4 Breastfeeding and formula feeding provide a baby with the same health benefits	28.2	1015 (96.5)	35.7	311 (94.2)	28.9	349 (97.2)	20.8	355 (97.8)
5 Formula helps babies sleep better	35.0	992 (94.3)	37.0	319 (96.7)	33.9	333 (92.8)	34.1	340 (93.7)
6 Formula is very like breastmilk	29.6	1007 (95.7)	32.4	315 (95.5)	32.4	343 (95.5)	24.4	349 (96.1)
7 Breastfeeding encourages better mother‐baby bonding^a^	73.8	1050 (99.8)	75.5	330 (100)	73.4	357 (99.4)	72.7	363 (100)
8 Formula keeps babies fuller for longer	55.1	1000 (95.1)	57.9	318 (96.4)	53.8	338 (94.2)	53.8	344 (94.8)
9 Breastfeeding in public is embarrassing	16.9	1048 (99.6)	15.6	327 (99.1)	19.2	359 (100)	15.7	362 (99.7)
10 Breastfed babies are healthier than formula fed babies^a^	29.5	1024 (97.3)	26.0	323 (97.9)	26.2	347 (96.7)	35.9	354 (97.5)
11 Formula feeding allows you to get your life back quicker	47.5	1036 (98.5)	43.1	327 (99.1)	45.9	351 (97.8)	53.1	358 (98.6)
12 Breastfeeding is old fashioned	2.9	1050 (99.8)	2.4	329 (99.7)	3.1	358 (99.7)	3.0	363 (100)
13 Breastfeeding helps you get your body shape back quicker^a^	52.7	1012 (96.2)	53.3	315 (95.5)	48.3	344 (95.8)	56.4	353 (97.2)
14 My partner prefers me not to breastfeed	5.0	1027 (97.6)	6.4	313 (94.8)	4.8	354 (98.6)	3.9	360 (99.2)
15 You should not feel pressurised to breastfeed	95.8	1050 (99.8)	96.7	329 (99.7)	95.0	358 (99.7)	95.9	363 (100)
16 Formula feeding means I can leave my baby with others	74.4	1048 (99.6)	73.9	329 (99.7)	71.4	357 (99.4)	77.9	362 (99.7)
17 There should be much more support to help women breastfeed successfully^a^	84.4	1040 (98.9)	83.3	329 (99.7)	83.2	352 (98.1)	86.6	359 (98.9)

^a^ Item reverse coded to calculate CMF positivity score.

**Table 4:** Belief factor names and factor loadings for belief statements about infant feeding (after rotation).

	Belief factors					
	Formula same or better	Formula keeps babies contented	Breastfeeding is not superior	Lifestyle advantages of formula	Breastfeeding is not appealing	Resistance to expectations to breastfeed
	*α* = 0.54	*α* = 0.71	*α* = 0.46	*α* = 0.54	*α* = 0.21	—
Belief statement	1	2	3	4	5	6
1 Formula feeding is the better choice if the mother plans to go back to work	0.443			**0.400**		
2 Breastfeeding is best for your baby^a^	**0.471**		0.466			
3 Formula‐fed babies grow better than breastfed babies	**0.385**	0.385	−0.362			
4 Breastfeeding and formula feeding provide a baby with the same health benefits	**0.762**					
5 Formula helps babies sleep better		**0.791**				
6 Formula is very like breastmilk	**0.677**					
7 Breastfeeding encourages better mother‐baby bonding^a^			**0.667**			
8 Formula keeps babies fuller for longer		**0.829**				
9 Breastfeeding in public is embarrassing					**0.329**	0.592
10 Breastfed babies are healthier than formula fed babies^a^	0.421		**0.573**			
11 Formula feeding allows you to get your life back quicker				**0.669**		
12 Breastfeeding is old fashioned					**0.730**	
13 Breastfeeding helps you get your body shape back quicker^a^			**0.617**			
14 My partner prefers me not to breastfeed					**0.660**	
15 You should not feel pressurised to breastfeed						**0.674**
16 Formula feeding means I can leave my baby with others				**0.739**		
17 There should be much more support to help women breastfeed successfully^a^	**0.362**					−0.448

*Note:* Loadings in bold indicate the belief factor that each belief statement was incorporated into. Cronbach's alpha coefficients (*α*) were calculated to evaluate the internal reliability of factors. Note Factor 6 ‘Resistance to expectations to breastfeed’ only had one item hence Cronbach's alpha could not be calculated.

^a^ Item reverse coded to calculate CMF positivity score.

No differences across SEP groups were found for the PCA‐derived belief factor ‘formula keeps babies contented’ (*p* = 0.526). There was a significant effect of SEP on the multivariate pattern of positive beliefs [Pillai's trace = 0.07, *F* (30, 1660) = 1.99, *p* = 0.001]. See Table 5 for full results. Women of lower SEP held the belief ‘formula feeding is the better choice if the mother plans to go back to work’ more strongly than women of medium SEP (*p* = 0.041) and higher SEP (*p* < 0.001). Women of lower SEP (*p* < 0.001) and medium SEP (*p* = 0.004) held the belief ‘breastfeeding and formula feeding provide a baby with the same health benefits’ more strongly than women of higher SEP. Compared to women of higher SEP, those of lower SEP held the belief ‘formula is very like breastmilk’ more strongly (*p* = 0.043) and the belief ‘breastfeeding is best for your baby’ less strongly (*p* < 0.001).

**Table 5:** Women's agreement with belief statements about infant feeding, by socioeconomic position (SEP).

	Lower SEP (*n* = 330)	Medium SEP (*n* = 359)	High SEP (*n* = 363)	*p*‐trend^a^
Formula feeding is the better choice if the mother plans to go back to work	0.95 (±0.90)	0.78 (±0.86)	0.66 (±0.82)	**< 0.001**
Breastfeeding is best for your baby^b^	0.47 (±0.75)	0.38 (±0.68)	0.26 (±0.59)	**0.001**
Formula fed babies grow better than breastfed babies	0.29 (±0.53)	0.33 (±0.58)	0.25 (±0.51)	0.222
Breastfeeding and formula feeding provide a baby with the same health benefits	0.85 (±0.92)	0.70 (±0.88)	0.48 (±0.81)	**< 0.001**
Formula is very like breastmilk	0.85 (±0.87)	0.82 (±0.89)	0.65 (±0.86)	**0.021**
Breastfeeding encourages better mother‐baby bonding^b^	0.40 (±0.74)	0.49 (±0.79)	0.45 (±0.78)	0.537
Breastfeeding in public is embarrassing	0.33 (±0.69)	0.41 (±0.77)	0.40 (±0.75)	0.606
Breastfed babies are healthier than formula fed babies^b^	1.24 (±0.84)	1.16 (±0.81)	1.05 (±0.87)	0.101
Formula feeding allows you to get your life back quicker	1.02 (±0.91)	1.02 (±0.94)	1.19 (±0.92)	0.221
Breastfeeding is old fashioned	0.11 (±0.37)	0.11 (±0.41)	0.09 (±0.37)	0.840
Breastfeeding helps you get your body shape back quicker^b^	0.76 (±0.87)	0.89 (±0.89)	0.71 (±0.87)	0.050
My partner prefers me not to breastfeed	0.22 (±0.53)	0.21 (±0.52)	0.14 (±0.44)	0.638
You should not feel pressurised to breastfeed	1.95 (±0.31)	1.93 (±0.34)	1.93 (±0.34)	0.613
Formula feeding means I can leave my baby with others	1.59 (±0.75)	1.56 (±0.77)	1.65 (±0.72)	0.476
There should be much more support to help women breastfeed successfully^b^	0.24 (±0.58)	0.25 (±0.58)	0.19 (±0.54)	0.193

*Note:* MANCOVA was conducted for 15 belief statements (belief statements from factor ‘Formula keeps babies contented’ were not included). Participants were asked to indicate ‘Disagree’, ‘Neither Agree nor Disagree’ or ‘Agree’ to belief statements. Answers were scored 0, 1 or 2 for statements expressing a positive belief about CMF. Reverse scoring (2, 1 or 0) was applied to statements showing positive beliefs about breastfeeding.

^a^ *p* value for trend derived using MANCOVA.

^b^ Item reverse coded.

No associations between CMF marketing score and CMF positivity score were found for the PCA‐derived belief factor ‘Formula keeps babies contented’ (*p* = 0.068) (Supporting Information S1).

## 4 Discussion

This survey of 1052 pregnant women and mothers in the UK explored women's self‐reported exposure to CMF marketing and their beliefs about CMF across SEP. Every participant reported exposure to CMF marketing, with women of different SEP (indexed using occupation) recalling exposure to a similar number of different types of CMF marketing. However, women of lower or middle SEP held stronger beliefs around CMF being the same or better than breastmilk. No association was found between the number of forms of CMF marketing women were exposed to and holding positive beliefs about CMF, but more sophisticated measures of marketing exposure are needed to fully explore associations between marketing exposure and beliefs.

Women reported frequent exposure to CMF advertising via a wide range of channels, including more traditional means such as TV and magazines as well as multiple digital channels. They also received targeted emails, texts and pop‐up advertisements and actively engaged with companies by signing up to their newsletters, ‘baby clubs’ and social media feeds. All these methods of marketing, while not individually listed, contravene the International Code for the Marketing of Breast‐milk Substitutes (the Code), which prohibits all direct‐to‐consumer advertising and promotion (World Health Organization 1981; World Health Organisation 2018). Legislation in the UK prohibits advertisements for infant formula labelled as suitable from birth, so the advertisements women saw would have been for follow‐on formula (suitable from 6 months) or toddler formula (suitable from 1 year). However, seeing formula for older children has been found to passively build brand belief and loyalty and influence infant feeding decisions (Rollins et al. 2023). Infant and follow‐on formula are sold in the UK in very similar packaging, and women have reported seeing advertisements for infant formula despite only being exposed to follow‐on formula advertising (Cattaneo et al. 2014; Conway et al. 2023a). Mothers in the UK have also described choosing CMF for new‐born infants based on the formula advertisements they had been exposed to over their lifetime (Conway et al. 2023b).

The multifaceted nature of CMF marketing strategies has been described by Rollins et al. (2023), who express particular concern over the growth of digital marketing as this extends companies' reach while circumventing regulations. Women in the current study reported exposure to marketing on a range of digital platforms, including YouTube, Facebook and Instagram, as well as engaging with CMF companies via company websites, following them on social media and signing up to receive further information supplied via ‘newsletters’. One in five women had signed up to company ‘baby clubs’, which are vehicles favoured by the CMF companies as they are effective for establishing and fostering relationships with customers without mentioning products (Hastings et al. 2020). Supermarkets also played a key role in promoting CMF, with a third of women reporting frequently seeing marketing or advertisements in store. Fewer women saw CMF advertising in hospitals or clinics compared to television or social media but seeing advertisements in these medical settings, and moreover receiving free CMF in hospitals, has been shown to be particularly impactful. Women in the US have described advertising and free samples in hospitals countering the advice they received about breastfeeding and undermining their commitment to establish breastfeeding in the early days (Parry et al. 2013).

No differences were found between women of different SEP in terms of the number of CMF marketing forms they had been exposed to. However, they may have been exposed to different types of marketing via these channels. Considering that digital marketing is becoming the dominant form of CMF promotion (World Health Organization 2022), alongside the fact that internet users in the UK exceed 92% of the population (Statista 2022), it is likely that all women across the socioeconomic range have been exposed to formula marketing. Targeted marketing has been described, with mothers who aspire to give their baby ‘a successful, middle class future’ receiving marketing about ‘research’ and a company's ‘advanced formulation’, while others are targeted with adverts showing contented giggling babies (Hastings et al. 2020).

The majority of women in this sample agreed with the statement ‘breastfeeding is best for your baby’; however, fewer agreed with the statements ‘breastfed babies are healthier than formula fed babies’. Some of these beliefs appear contradictory, reflecting the inherent complexity in capturing beliefs, particularly via a survey. In qualitative studies, women explaining their infant feeding decisions describe the complex interaction of multiple factors, including beliefs about health, practical considerations, cultural norms and perceived social and family support (Radzyminski and Callister 2016). The lack of association observed between the number of forms of CMF marketing women were exposed to, and their beliefs about CMF is not surprising given the relatively crude assessments of marketing exposure and beliefs used in the current survey, compared to the complexity of both of these phenomena. Still, some differences between the beliefs held by women of different SEP groups were found, which may contribute to our understanding of the lower rates of breastfeeding observed among women of lower SEP in the UK. Both the lower and middle SEP groups were more likely to hold certain positive beliefs about CMF compared to their higher SEP counterparts. Structural and wider environmental factors have previously been the focus of discussions about socioeconomic inequalities in breastfeeding rates, including inadequate maternity protection and lack of support for breastfeeding. Even if women experience similar exposure to CMF marketing, it has been suggested that the way this is interpreted may differ as it is shaped by their experiences in the healthcare system, employment system, family and community (Rollins et al. 2016).

The high level of exposure to CMF marketing reported here is in line with WHO reports, which point to pervasive and manipulative methods being used by the multibillion‐dollar CMF industry to sell CMF to families around the world (Piwoz and Huffman 2015; World Health Organization and the United Nations Children's Fund UNICEF 2022). Our results highlight a particular need to tackle marketing that misleads parents by suggesting CMF offers the same benefits as breastmilk (Rollins et al. 2023). CMF companies have been criticised for exploiting the vulnerabilities of new parents by pitching CMF in ways that seem reassuring, sympathetic and non‐judgemental while conveying the message that CMF is convenient, compatible with modern lifestyles and equivalent or even superior to breastmilk (Hastings et al. 2020; Mejia et al. 2016). Most countries, including the UK, have legislation in place that aims to protect the public from inappropriate and potentially harmful CMF marketing, for example, by restricting the use of nutrition and health claims on infant formula (McFadden et al. 2016; World Health Organization and the United Nations Children's Fund UNICEF 2022). However, legislation is outdated, as it does not address implied claims about similarities between CMF and breastmilk or tackle the multifaceted digital marketing strategies used (Pérez‐Escamilla et al. 2023).

This is the first study to explore the association between the SEP of pregnant women and mothers in the UK, their self‐reported exposure to CMF marketing and the extent to which they hold positive beliefs towards CMF. However, there were several limitations. SEP was defined by a single indicator, occupational group, as this was used for quota sampling and allowed comparison between three approximately equal‐sized groups. However, SEP operates at various levels, including individual, household and neighbourhood and if participants had been sampled using an alternative indicator (such as income or education) results may have been different. Quota sampling by occupational group facilitated inclusion of women across the socioeconomic spectrum, which is a strength of this study, and trends in education, household income and maternal age between the three SEP groups suggest it was a suitable choice (Table 1). As mentioned, the scale for measuring marketing exposure was rather crude. Another limitation is that infrequent or subconscious exposure to marketing was not accounted for, as women were asked to report where they had ‘frequently’ seen advertisements, which means true exposure may have been underestimated. Future studies using emerging technologies to capture real time exposure to marketing, particularly digital marketing would provide a better understanding of the nature of women's true exposure to CMF marketing. Furthermore, participants were recruited via online research panels and advertising, which may have resulted in overestimation of exposure to digital CMF marketing due to selection bias. Finally, the survey was conducted during the COVID‐19 pandemic, which may have impacted results although data suggests differences at a population level were small (Quigley et al. 2023). Research in the UK has shown that home confinement acted as a facilitating factor for breastfeeding for some mothers, while isolation, anxiety about the safety of breastfeeding and limited support acted as barriers for others (Brown and Shenker 2021). Also, the increase in screen time that has been documented during COVID‐19 (Trott et al. 2022) may have raised mothers' levels of exposure to CMF marketing.

In conclusion, pregnant women and new mothers in the UK recalled widespread exposure to CMF marketing via multiple channels. Positive beliefs about CMF were common, and women of lower and middle SEP were more likely to believe that CMF was the same or better than breastfeeding, compared to women of higher SEP. There is a need to update and enforce legislation to prevent misleading marketing on and offline that portrays CMF as equivalent or superior to breastmilk – this would support all families to make informed choices about infant feeding, but particularly those of lower and middle SEP. To effectively reduce inequalities in breastfeeding rates, wider‐reaching policies are essential which address factors such as maternity employment protection and support for breastfeeding.

## Author Contributions

R.C., M.A. and F.S. designed the analytical approach. M.A. and F.S. carried out the analysis. R.C., M.A. and F.S. wrote the paper. All authors reviewed and approved the final manuscript.

## Conflicts of Interest

The authors declare no conflicts of interest.

## Supporting information

Supporting information.

## Data Availability

The data that support the findings of this study are available on request from the corresponding author. The data are not publicly available due to privacy or ethical restrictions.
