# Supplementary material for: Cross‐Sectional Associations Between Exposure to Commercial Milk Formula Marketing, Beliefs About Its Use, and Socioeconomic Position Among Pregnant Women and Mothers in the UK
Source: Matern Child Nutr. 2025 Mar 24;21(3):e70022. doi: 10.1111/mcn.70022 (PMC12150155; doi:10.1111/mcn.70022)
Supplement: Supplementary file 1 — Supporting information. [file MCN-21-e70022-s001.docx]

**Supplementary file for Cross-sectional associations between exposure to commercial milk formula marketing, beliefs about its use, and socioeconomic position among pregnant women and mothers in the UK**

1. Survey extract – page 1
2. Socioeconomic Position (SEP) classification by occupation - page 18
3. Results of regression analysis – page 19
4. Correlation between Socioeconomic Position (SEP) and income – page 19
5. **Survey Extract**

**Survey screener**

1. **Do you or does any member of your family or close friends work for any of the following companies?**
2. A market research company/ marketing company
3. A company that makes infant formula milk
4. A bank
5. An advertising agency
6. A company that works with infant formula milk companies
7. None of these
8. Don’t know
9. **How old were you at your last birthday?**
10. Under 18
11. 18-24
12. 25-29
13. 30-34
14. 35-39
15. 40-44
16. 45-49
17. Over 50

Write in actual age ___________years

1. **Can you speak, read, write and understand English fluently?**
2. Yes
3. No
4. **Which of the following apply to you?**
5. I am pregnant (more than 3 months)
6. I have a baby aged 0-18 months
7. None of the above
8. **Which of the following have you done in the past twelve months? You can say as many as apply.**
9. Been to a movie
10. Been to a job interview
11. Eaten at a restaurant
12. Attended a group discussion, or been interviewed for research related to mothers and babies
13. Been to a wedding
14. None of these
15. **How do you plan to feed your baby in the first two weeks?**
16. Breastfeeding only
17. Formula feeding only
18. Both breastfeeding and formula feeding from birth
19. Breastfeeding first and then switch to formula feeding
20. Breastfeeding first and then breastfeeding and giving formula
21. Breastfeeding and formula feeding first and then switch to formula feeding
22. Breastfeeding and formula feeding first and then switch to breastfeeding
23. I haven’t planned yet/ don’t know
24. **Is this the first baby that you have given birth to, that you will feed?**
25. Yes
26. No
27. **How old is your youngest baby?**
28. Younger than one month
29. Between one and two months
30. 2 - 3 months
31. 4 - 6 months
32. 7 - 9 months
33. 10 -12 months
34. 13 -18 months
35. Over 18 months

**9.** **How are you currently feeding your youngest baby?**

1. Breastfeeding only since birth
2. Formula feeding only since birth
3. Both breastfeeding and formula feeding from birth
4. Breastfed first and now I am formula feeding
5. Breastfed first and now I am breastfeeding and giving formula
6. Breastfeeding and formula feeding first, and now giving formula
7. Breastfed and formula feeding first and now breastfeeding only

**10. Do you or your baby have any medical conditions (e.g. infectious diseases, specific medications) that prevents, or would prevent you from breastfeeding?**

1. Yes
2. No
3. Don’t know

**11. How old was your youngest baby when you started formula feeding**

1. A day or less
2. 2-6 days
3. 1-2 weeks
4. 3-4 weeks
5. 5-6 weeks
6. 7-8 weeks
7. 2+ months (including 2 months)
8. 3+ months (including 3 months
9. 4+ months (including 4 months)
10. 5+ months (including 5 months)
11. 6- 8 months
12. 9-12 months
13. More than 12 months

**12. How many weeks pregnant are you?**

1. 0-13 weeks (first trimester)
2. 14-26 weeks (second trimester)
3. 27-42 weeks (third trimester)
4. I am not sure

**13.** **To which one of the following occupational groups does the main income earner in your household belong, or which groups fits best. The main income earner is the person in your household with the largest income.**

**If the main income earner is retired and has an occupational pension, please answer for the occupation which was their main career. If the main income earner is not in paid employment but has been out of work for less than 6 months, please answer for their most recent occupation.**

1. Professionals, very senior business or very senior civil servants (e.g. Doctor, solicitor, barrister, CEOs, Managing Director)
2. Middle Management executives in large organisations, principal officers in local government and civil services, top managers or owners of small businesses (e.g. middle manager, teacher, nurse, police officer)
3. Supervisory or junior manager, administrator or professional (e.g. junior manager, students living away from home, clerical/ office worker, supervisor)
4. Skilled manual worker (e.g. Foreman, agricultural worker, plumber, bricklayer, hairdresser)
5. Other types of manual workers & apprentices (e.g. labourer, shop assistant, fisherman, apprentice)
6. Casual worker or dependent on a state pension
7. Unemployed or dependent on unemployment/ other benefits

**14. What is your annual household income, before tax and deductions?**

1. Less than £10,000
2. £10,000 - £19,999
3. £20,000 – £29,999
4. £30,000 –£39,999
5. £40,000 – £49,999
6. £50,000 – £59,999
7. £60,000 –£69,999
8. £70,000- £79,999
9. £80,000-£89,999
10. £90,000- £99,999
11. £100,000 or more
12. Prefer not to answer

**15. How many other (than your youngest baby under 18 months) children, if any, do you have?**

1. None
2. One
3. Two
4. Three
5. Four
6. Five
7. Six
8. Seven
9. Eight
10. Nine
11. Ten or more
12. **What is your marital status?**

1. Single
2. Married
3. Cohabiting
4. Divorced/separated
5. Widowed
6. **Which of these ethnic groups best describes you?**
   1. White, Scottish/English/Welsh/Northern Irish/British
   2. White, Irish
   3. White, gypsy or Irish traveller
   4. Any other White background
   5. Mixed/ multi, white and black Caribbean
   6. Mixed/multi white and black African
   7. Mixed/multi white and Asian
   8. Any other mixed/ multi-ethnic
   9. Asian/ Asian British, Indian
   10. Asian/ Asian British, Pakistani
   11. Asian/ Asian British, Bangladeshi
   12. Asian/ Asian British Chinese
   13. Any other Asian background
   14. Black/ black British African
   15. Black/ black British Caribbean
   16. Other black/African/ Caribbean
   17. Arab
   18. Any other ethnic group
   19. Prefer not to say/refused

**Main survey**

**1. What is the highest level of education that you have completed?**

**e.g. school/ college/ university?**

1. Primary (up to the age of 11)
2. Secondary (up to the age of 16)
3. Secondary (up to the age of 18)
4. Higher education – university or college (over age of 18)
5. Post-graduate education (e.g. Masters, PhD)
6. Other
7. No education

**2. Are you currently working?**

1. I work full time (30 hours + per week)
2. I work part time (8-29 hours per week)
3. I work part time (less than 8 hours per week)
4. I am a housewife at home/ full time mother
5. I am on maternity leave
6. I am unemployed / looking for a job
7. I am in full time education

**3. Are you receiving/will be receiving any maternity pay or benefits?**

1. Yes, just statutory maternity benefit
2. No, I don’t receive any formal maternity benefit
3. Yes, my employer pays me more than the statutory rate of pay/ gives me maternity benefits

**4. Are you taking/ planning to take any maternity leave?**

1. Yes
2. No
3. Not sure

**5.** **How long are you taking/ planning to take for maternity leave?**

1. Less than 1 month
2. 1-2 months
3. 3-4 months
4. 5-6 months
5. 7-9 months
6. 10-12 months
7. More than 12 months

**6. Which suburb or district do you live in?**

| **London** | **Glasgow** |
| --- | --- |
|  |  |

**7. Did you give birth/ Do you plan to give birth in a hospital?**

1. Yes, at public hospital
2. Yes, at private hospital
3. No
4. Don’t know/Not sure

**8. Which hospital did you give birth in / do you plan to give birth in?**

**London**

| **Code** | **Statement list** |  |
| --- | --- | --- |
| 1 | Barnet Hospital |  |
| 2 | Central Middlesex Hospital |  |
| 3 | Charing Cross |  |
| 4 | Chelsea and Westminster Hospital |  |
| 5 | Croydon University Hospital |  |
| 6 | Ealing Hospital |  |
| 7 | Fitzrovia Suite at University College Hospital |  |
| 8 | Guy’s Hospital |  |
| 9 | Hillingdon Hospital |  |
| 10 | Homerton Hospital |  |
| 11 | King’s College Hospital |  |
| 12 | King George Hospital |  |
| 13 | Kingston Hospital |  |
| 14 | North Middlesex University Hospital |  |
| 15 | Northwick Park Hospital |  |
| 16 | Princess Royal University Hospital |  |
| 17 | Queen Charlottes & Chelsea Hospital |  |
| 18 | Queen Elizabeth Hospital, Kings Lynn |  |
| 19 | Queen’s Hospital |  |
| 20 | Royal Free Hospital |  |
| 21 | Royal London Hospital |  |
| 22 | Sir Stanley Clayton Ward at Queen Charlotte’s & Chelsea |  |
| 23 | St. George’s Hospital |  |
| 24 | St. Helier Hospital |  |
| 25 | St. Mary’s Hospital London |  |
| 26 | St. Thomas’ Hospital |  |
| 27 | The Kensington Wing at Chelsea and Westminster |  |
| 28 | The Lindo Wing at St. Mary’s Hospital |  |
| 29 | The Portland Hospital |  |
| 30 | University College Hospital |  |
| 31 | University Hospital Lewisham |  |
| 32 | West Middlesex University Hospital |  |
| 33 | Westminster Maternity Suite at Guy’s and St. Thomas’ |  |
| 34 | Whipps Cross Hospital |  |
| 35 | Whittington Hospital |  |

**Scotland**

| **Code** | **Statement list** |  |
| --- | --- | --- |
| 36 | Aberdeen Maternity Hospital |  |
| 37 | Arbroath Infirmary |  |
| 38 | Balfour Hospital, Orkney |  |
| 39 | Belford Hospital |  |
| 40 | BMI Carrick Green Hospital |  |
| 41 | BMI King’s Park Hospital |  |
| 42 | BMI Ross Green Hospital |  |
| 43 | Borders General Hospital, Melrose |  |
| 44 | Caithness General Hospital |  |
| 45 | Cambeltown Hospital |  |
| 46 | Cowal Community Hospital |  |
| 47 | Dumfries and Galloway Royal Infirmary |  |
| 48 | Galloway Community Hospital |  |
| 49 | Gilbert Bain Hospital |  |
| 50 | Glasgow Hospital, Nuffield |  |
| 51 | Inverclyde Royal Hospital |  |
| 52 | Islay Hospital |  |
| 53 | Isle of Arran War Memorial Hospital |  |
| 54 | Lorn and Islands District General Hospital |  |
| 55 | Mackinnon Memorial Hospital |  |
| 56 | Mid Argyll Hospital |  |
| 57 | Moffat Hospital |  |
| 58 | Montrose Royal Infirmary |  |
| 59 | Ninewells Hospital |  |
| 60 | Perth Royal Infirmary |  |
| 61 | Peterhead Community Hospital |  |
| 62 | Royal Alexandra Hospital |  |
| 63 | Royal Infirmary of Edinburgh |  |
| 64 | Uist and Barra Hospital |  |
| 65 | University Hospital Ayr |  |
| 66 | University Hospital Crosshouse |  |
| 67 | Vale of Leven Hospital |  |
| 68 | Victoria Hospital, Bute |  |
| 69 | Victoria Hospital, Kirkcaldy |  |

**Manchester**

| **Code** | **Statement list** |  |
| --- | --- | --- |
| 70 | BMI The Alexandra Hospital, Cheadle |  |
| 71 | Leigh Infirmary |  |
| 72 | Manchester Royal Infirmary |  |
| 73 | North Manchester General Hospital |  |
| 74 | Rochdale Infirmary |  |
| 75 | Royal Albert Edward Infirmary |  |
| 76 | Royal Bolton Hospital |  |
| 77 | Royal Oldham Hospital |  |
| 78 | Saint Mary’s Hospital |  |
| 79 | Salford Royal Hospital |  |
| 80 | Spire Manchester Hospital |  |
| 81 | Stepping Hill Hospital |  |
| 82 | Tameside & Glossop Integrated Care NHS Foundation Trust |  |
| 83 | Trafford General Hospital |  |
| 84 | Withington Hospital |  |
| 85 | Wythenshawe Hospital |  |
| 80 | Other - please specify |  |

**9. Was your youngest baby delivered by caesarean section** /**Do you plan to have your baby by caesarean section?**

1. Yes
2. No
3. Don’t know/Not sure

________________________________(Write in)

**FEEDING YOUR BABY**

**Thank you for telling us a bit about yourself, we would now like to ask you some questions about how you feed your youngest baby (under 18 months).**

**10. How long did you breastfeed your baby for, before you switched to formula feeding?**

1. A day or less
2. 2-6 days
3. 1-2 weeks
4. 3-4 weeks
5. 5-6 weeks
6. 7-8 weeks
7. 2+ months (including 2 months)
8. 3+ months (including 3 months
9. 4+ months (including 4 months)
10. 5+ months (including 5 months)
11. 6- 8 months
12. 9-12 months
13. More than 12 months

**11. Which of the following applies to you?**

1. I introduced formula before I stopped breastfeeding
2. I moved from breastfeeding only to feeding formula only

**12. You mentioned earlier in the survey that you ………** **Is this how you originally planned to feed your baby?**

- 1. Yes
  2. No
  3. I didn’t have a set plan for feeding my baby

**13. Overall, how does this differ from your original plan for feeding your baby?**

1. I have breastfed more/ for longer than I expected
2. I have fed formula more/ for longer than I expected
3. I have not breastfed at all
4. I have not fed formula at all
5. Other _______________(Write in)

**14. Do you intend to change the type of milk that you feed your baby?**

1. Yes, I will be introducing a formula milk alongside breast milk
2. Yes, I will be changing from only breastmilk to formula only
3. Yes , I intend to feed cows, goats, or other milks
4. Yes, I intend to switch to breastmilk only
5. I intend to stop feeding milk to my baby
6. I haven’t decided yet
7. Other
8. I will be feeding my baby in the same way

**15. What type of formula will you be feeding your baby?**

1. Infant formula (Stage 1)
2. Follow on formula (Stage 2)
3. Growing up formula (Stage 3 or 4)
4. A specialist formula e.g. for allergies
5. Other formula

**16. Have you ever breastfed your youngest baby?**

1. Yes
2. No

**INFORMATION ABOUT INFANT FEEDING**

**FACTORS INFLUENCING FEEDING**

**KNOWLEDGE AND USE OF FORMULA BRANDS**

**ATTITUDES TOWARDS INFANT FEEDING PRACTICES**

**39. In the next question, we want to explore your attitudes towards feeding your baby. We are very interested in your opinions and experiences, there are no right or wrong answers.**

**Please can you say whether you agree, disagree or neither agree nor disagree with each statement that I am going to read out.**

|  | **1**  **Disagree** | **2**  **Neither agree nor disagree** | **3**  **Agree** | **4**  **Don’t know/ N/A** |
| --- | --- | --- | --- | --- |
| 1. Formula feeding is the better choice if the mother plans to go back to work |  |  |  |  |
| 1. Breastfeeding is best for your baby |  |  |  |  |
| 1. Formula fed babies grow better than breastfed babies |  |  |  |  |
| 1. Breastfeeding and formula feeding provide a baby with the same health benefits |  |  |  |  |
| 1. Formula helps babies sleep better |  |  |  |  |
| 1. Formula is very like breastmilk |  |  |  |  |
| 1. Breastfeeding encourages better mother-baby bonding |  |  |  |  |
| 1. Formula keeps babies fuller for longer |  |  |  |  |
| 1. Breastfeeding in public is embarrassing |  |  |  |  |
| 1. Breastfed babies are healthier than formula fed babies |  |  |  |  |
| 1. Formula feeding allows you to get your life back quicker |  |  |  |  |
| 1. Breastfeeding is old fashioned |  |  |  |  |
| 1. Breastfeeding helps you get your body shape back quicker |  |  |  |  |
| 1. My partner prefers me not to breastfeed |  |  |  |  |
| 1. You shouldn’t feel pressurised to breastfeed |  |  |  |  |
| 1. Formula feeding means I can leave my baby with others |  |  |  |  |
| 1. There should be much more support to help women breastfeed successfully |  |  |  |  |

**ADVERTISING AND MARKETING**

**40. In the past year have you seen or heard any marketing or advertising for formula milk?** **This could include information or promotions on formula milk, YouTube or other videos, sponsored websites, Facebook or other social media sites, chat rooms or forums, as well as TV, radio, billboard advertising etc.**

1. Yes
2. No
3. Don’t Know

**41. Thinking about the last piece of marketing or advertising that you saw or heard for formula milk, can you describe what it was saying/ telling you?**

Write in ____________________________________________________

**42. And where did you see or hear this advert?**

1. TV
2. Radio
3. YouTube
4. Company website e.g. a specific brand website
5. Social media e.g. Facebook, Instagram, celebrity or mummy blogger
6. Online discussion forum or chat room
7. Another website
8. Mother’s club or group online
9. In an email
10. In the post
11. A hospital or clinic
12. Magazine or newspaper
13. Billboard e.g. on the highway or roadside
14. Supermarket/shop/market – in store
15. Supermarket/ shop - online
16. Other (please write in _________________)

**43. What type of formula milk was this advertising for**

1. Infant formula (1)
2. Follow-on formula (2)
3. Toddler milk (3)
4. Growing up milk (3/4)
5. Specialised milks for babies with allergies
6. Other specialised milks? e.g. for sleep/ colic / hungry babies
7. Don’t remember/ Not sure

**44. In which of these locations have you frequently seen any type of marketing or advertising for formula brands?**

1. TV
2. Radio
3. YouTube
4. Company website e.g. a specific brand website
5. Social media e.g. Facebook, Instagram, celebrity or mummy blogger
6. Online discussion forum or chat room
7. Another website
8. Mother’s club or group online
9. In an email
10. In the post
11. A hospital or clinic
12. Magazine or newspaper
13. Billboard e.g. on the highway or roadside
14. Supermarket/shop/market – in store
15. Supermarket/ shop - online
16. Other (please write in _________________)

**45. In the past year have you seen or heard any advertising about breastfeeding? When we say ‘advertising’, this could include information on breastfeeding videos, sponsored websites, social media content and chat rooms as well as TV, radio, billboard advertising etc.**

1. Yes
2. No
3. Don’t know

**46. Where did you see or hear this advert?**

1. TV
2. Radio
3. YouTube
4. Company website e.g. a specific brand website
5. Social media e.g. Facebook, Instagram, celebrity or mummy blogger
6. Online discussion forum or chat room
7. Another website
8. Mother’s club or group online
9. In an email
10. In the post
11. A hospital or clinic
12. Magazine or newspaper
13. Billboard e.g. on the highway or roadside
14. Supermarket/shop/market – in store
15. Supermarket/ shop - online
16. Other (please write in _________________)

**47. Please tell me, have you ever received any of the following?**

|  | **1**  **Yes** | **2**  **No** | **3**  **Don’t know / N/A** |
| --- | --- | --- | --- |
| 1. Information or contact from a formula company by email, post, phone, or text message that you haven’t requested |  |  |  |
| 2. Promotion for formula discount |  |  |  |
| 3. Free samples of formula milk in hospital |  |  |  |
| 4. Free sample of formula milk outside hospital |  |  |  |
| 5. Free bottles or teats |  |  |  |
| 6. Any other gifts from a formula company e.g. a toy, bag or clothing |  |  |  |
| 7. An invite to join a ‘baby club’ run by a formula company |  |  |  |
| 8. An invite to a competition from a formula company or from a shop |  |  |  |
| 10. An invite from a formula company to participate in research/ survey/ interview |  |  |  |
| 12. Received a pop-up advert on Facebook or other social media |  |  |  |

**48. Do you do/ have you done any of the following?**

1. Follow a formula company on social media
2. Use an app from a formula company
3. Participate in any baby competitions run by formula companies
4. Use any information from formula companies
5. Registered for updates/ newsletter from a formula company
6. Signed up/ registered for a baby club run by a formula company
7. Followed a person on Instagram or Facebook for information on formula feeding
8. None of these

**49. Thinking of all the adverts that you have seen, can you remember whether any of these adverts featured any of the following messages?**

**50. If you were looking to choose a brand of formula, which of the following messages would be most appealing to you?**

|  | **Q49 MESSAGES SEEN** | **Q50 MOST APPEALING** |
| --- | --- | --- |
| 1. Improves babies sleep |  |  |
| 2. Improves babies health |  |  |
| 3. Improves babies brain development |  |  |
| 4. Promotes healthy weight gain |  |  |
| 5. Is similar to breastmilk |  |  |
| 7. Easy to digest |  |  |
| 8. Good for allergies |  |  |
| 10. Keeps babies fuller for longer |  |  |
| 13. Breastmilk is best |  |  |
| 14.Helps babies/children grow taller |  |  |
| 15. Increases immunity |  |  |
| 16.Contains essential nutrients |  |  |
| 1. Contains DHA |  |  |
| 1. Enriched with iron |  |  |
| 1. None of these |  |  |

**51a. I am now going to read out some statements about advertising for formula milk, and packaging of formula milk. For each, please can you say whether you agree or disagree or neither agree nor disagree.**

|  | **Disagree** | **Neither agree nor disagree** | **Agree** |
| --- | --- | --- | --- |
| 1. Formula ads help me to make decisions about how I will feed my baby |  |  |  |
| 1. I think that formula ads are trustworthy |  |  |  |
| 1. Formula ads are confusing |  |  |  |
| 1. Formula ads suggest that bottle feeding formula is just as good as breast feeding |  |  |  |
| 1. When I look at tins of formula in the shop, I am not sure which is the right one to buy |  |  |  |
| 1. I think that the more expensive brands of formula are better for your baby |  |  |  |

**51b. I am going to read out a number of statements about the labels on formula milk tins, please say for each, whether you agree, disagree or neither agree not disagree.**

|  | **Disagree** | **Neither agree nor disagree** | **Agree** |
| --- | --- | --- | --- |
| 1. Tins of formula milk should state the difference between breastmilk and formula milk |  |  |  |
| 1. Tins of formula should have labels to warn you when they have high levels of sugar. |  |  |  |
| 1. It would be good if you could buy generic ‘no brand’ formula milks |  |  |  |
| 1. I would like tins of milk to have information that only comes from Ministry of Health |  |  |  |
| 1. I would like to have a clearer understanding of what is in formula milk |  |  |  |
| 6. I rely on the information on formula tins to decide which one to buy |  |  |  |

**2. Socioeconomic position (SEP) classification by occupation**

| HIGH SEP | Professionals, very senior business, or very senior civil servants (e.g., Doctor, solicitor, barrister, CEOs, Managing Director) |
| --- | --- |
|  | Middle Management executives in large organisations, principal officers in local government and civil services, top managers, or owners of small businesses (e.g., middle manager, teacher, nurse, police officer) |
| MEDIUM SEP | Supervisory or junior manager, administrator or professional (e.g., junior manager, students living away from home, clerical/ office worker, supervisor) |
|  | Skilled manual worker (e.g., Foreman, agricultural worker, plumber, bricklayer, hairdresser) |
| LOW SEP | Other types of manual workers & apprentices (e.g., labourer, shop assistant, fisherman, apprentice) |
|  | Casual worker or dependent on a state pension |
|  | Unemployed or dependent on unemployment/other benefits |

*Note.* The main income earner refers to the person in the household with the highest income. In cases of retirement, responses were given according to main career and, in cases of unemployment lasting less than six months, responses were given according to most recent occupation.

**3. Results of regression analysis**

A regression analysis was conducted to explore the association between CMF marketing score and CMF positivity score for the PCA-derived belief factor “Formula keeps babies contended”. The regression model was not significant, ΔR^2^ = 0.01, F(5, 966) = 2.07, p=.068.

**4. Correlation between Socioeconomic Position (SEP) and income**

SEP was found to be significantly positively correlated with income, *rs*(1017)= .66, p<.001.
